# Comparison of choroidal thickness measurements between spectral domain optical coherence tomography and swept source optical coherence tomography in children

**DOI:** 10.1038/s41598-021-92980-9

**Published:** 2021-07-02

**Authors:** Chun On Lee, Xiujuan Zhang, Nan Yuan, Shumin Tang, Li Jia Chen, Carol Y. Cheung, Jason C. Yam

**Affiliations:** 1 Department of Ophthalmology and Visual Sciences, The Chinese University of Hong Kong, Hong Kong SAR, China; 2 Department of Ophthalmology and Visual Sciences, Prince of Wales Hospital, Hong Kong SAR, China; 3 Hong Kong Eye Hospital, Hong Kong SAR, China

**Keywords:** Eye diseases, Medical imaging

## Abstract

Choroidal thickness is associated with many ocular conditions, interchangeability among different generations of optical coherence tomography is therefore important for both research purpose and clinical application. Hence, we compared choroidal thickness measurements between spectral-domain optical coherence tomography (SD-OCT) and swept-source optical coherence tomography (SS-OCT) in healthy paediatric eyes. A total of 114 children from the population-based Hong Kong Children Eye Study with mean age of 7.38 ± 0.82 years were included. Choroidal thickness of the right eye was measured by both devices. The central foveal choroidal thickness (CFCT) measured by SD-OCT and SS-OCT was 273.24 ± 54.29 μm and 251.84 ± 47.12 μm respectively. Inter-device correlation coefficient was 0.840 (95% CI 0.616–0.918). However, choroidal thickness obtained by SD-OCT was significantly thicker than that measured by SS-OCT with a mean difference of 21.40 ± 33.13 μm (*P* < 0.001). Bland–Altman limit of agreement on the relative difference scale for SD-OCT/SS-OCT was 86.33 μm. Validated conversion equation for translating SD-OCT CFCT measurement into SS-OCT was SS-OCT = 35.261 + 0.810 × SD-OCT. In conclusion, intra-class correlation coefficient (ICC) shows an acceptable agreement between SD-OCT and SS-OCT, however, there was a significant inter-device difference of choroidal thickness measurements in normal children eyes. Therefore, the measurements are not interchangeable.

## Introduction

The highly-vascularised choroid is the middle tunic of the eye, supplying nearly 90% of ocular artery blood flow. It is crucial in thermoregulation, regulation of intraocular pressure (IOP) and drainage of aqueous humor. Longitudinal studies of choroid in children suggest its role in eye development, including emmetropisation, growth factor secretion and scleral growth modulation^1,2^.

Associations of choroidal thickness as measured by optical coherence tomography (OCT) have been reported for retinal pathologies, including myopia. Consistent evidence has shown the close link between myopia and choroidal thinning in both children and adults^1–4^. Therefore, choroidal changes since childhood may be an early indictor in myopic development. Accurate and reliable monitoring of temporal changes in choroidal thickness may help in studying the role of choroid in myopia.

Using the enhanced depth imaging (EDI) mode with spectral-domain optical coherence tomography (SD-OCT), peak sensitivity of measurements is positioned at the inner sclera, enabling visualisation of deeper tissue layers including choroidal structure^5–8^. Whereas swept-source optical coherence tomography (SS-OCT), the newer generation of OCT, uses a tunable laser beam to sweep across various layers with a single photodiode detector. By employing light beam with a longer wavelength, SS-OCT could provide a higher quality of choroid imaging by having less signal noise and better penetration in deeper structures^5–7^.

Owing to the advantage of greater scanning depth and availability of commercialised SS-OCT, many researchers have switched from SD-OCT to SS-OCT in assessing deep structures like choroid. Nevertheless, it is still questionable whether measurements made by SD-OCT and SS-OCT images are interchangeable. Many studies comparing the two commonly used generations of OCTs (SD-OCT and SS-OCT) have been focusing on either normal^9–16^ or diseased^12,15,17,18^ adult eyes. However, little is known about the inter-device variability in choroidal thickness measurements in children eyes.

However, such information may have been obtained from different generations of OCTs across the years, determining the interchangeability among two generations of OCTs are therefore of significant use. Furthermore, valid conversion equations would enable studies to pool data from two generations of OCTs.

In view of the potential utilisation of choroidal thickness data from different generations of OCTs to monitor choroidal thickness changes for research purpose or clinical application, we would like to compare the choroidal thickness measurements between SD-OCT and SS-OCT in school children in the Hong Kong Children Eye Study (HKCES). Conversion equations enable longitudinal trials to pool data from two generations of OCTs to monitor changes across the years.

## Materials and methods

### Study population

Data for this analysis were derived from the Hong Kong Children Eye Study (HKCES), a population-based cohort study of eye conditions in children aged 6 to 8 years^19–23^. Study subjects were recruited consecutively from November to December 2015. All underwent the comprehensive ophthalmic examination, physical examination and standardised questionnaire at the Chinese University of Hong Kong Eye Centre.

Informed consent was obtained from all subjects and their legal guardians before participation. The study procedure was performed in accordance with the Declaration of Helsinki and the protocol was approved by the Ethic Committee Board of the Chinese University of Hong Kong. Subjects with ocular diseases (except myopia and hypermetropia), congenital eye malformations, prior eye trauma, previous eye surgery or incapable to complete OCT scan, were excluded. In this study, only right eyes were analysed in view of the high correlation between both eyes.

### Ophthalmic and physical examinations

Each child’s cornea, anterior chamber, iris, pupil reflex, crystalline lens, anterior vitreous and eyelids were examined by an ophthalmologist using Haag-Streit slit-lamp (Koeniz, Switzerland) to exclude underlying ocular diseases. Pre- and post-cycloplegic refractive status were measured by autorefractor (Nidek ARK-510A, Gamagori, Japan). Two rounds of cycloplegic agents (1% cyclopentolate (Alcon, Belgium) and 1% tropicamide (Santen, Japan)) were applied at 10 min apart. Post-cycloplegic spherocylindrical autorefraction was measured at least 30 min after the last drop of cycloplegic agent.

Body height and weight were measured by a professional integrated set (seca, Hamburg, Germany). Waist and head circumferences were measured using a tape measurer.

### Choroidal imaging

For SD-OCT, Heidelberg Eye Explorer, Version 1.6.1.0 (Heidelberg Engineering, Heidelberg, Germany) was employed for choroidal imaging. The system adopted a volume scan pattern (25° × 30°; 32 total B-scans) centred on the fovea. Each B-scan in the volume was a composite average of 35 individual line-scan images and the 45-degree cross-line scan pattern was used. With the EDI protocol, contrast of the choroid was enhanced^24^. All images were inspected, and choroidal layer was manually segmented using a MATLAB software by a well-trained examiner (C.O.L.). In the segmentation for each radial scan, the fovea was denoted by a dot and 31 points were plotted for both the upper and lower boundaries of choroidal layer.

For SS-OCT, Triton DRI-OCT, Version 1.1.5.47004 (Topcon, Inc, Tokyo, Japan) was used. A 1050-nm-wavelength swept-laser was used with a scanning speed of 100,000 A-scans per second. Image was obtained in 12-line radial scan pattern with a resolution of 1059 × 400. An average of 32 overlapped consecutive scans, covering an area of 12 mm × 9 mm, was acquired for each image. Choroidal layer was segmented using a built-in software. All segmented images were further inspected and manually modified to ensure accuracy.

**Figure 1:**
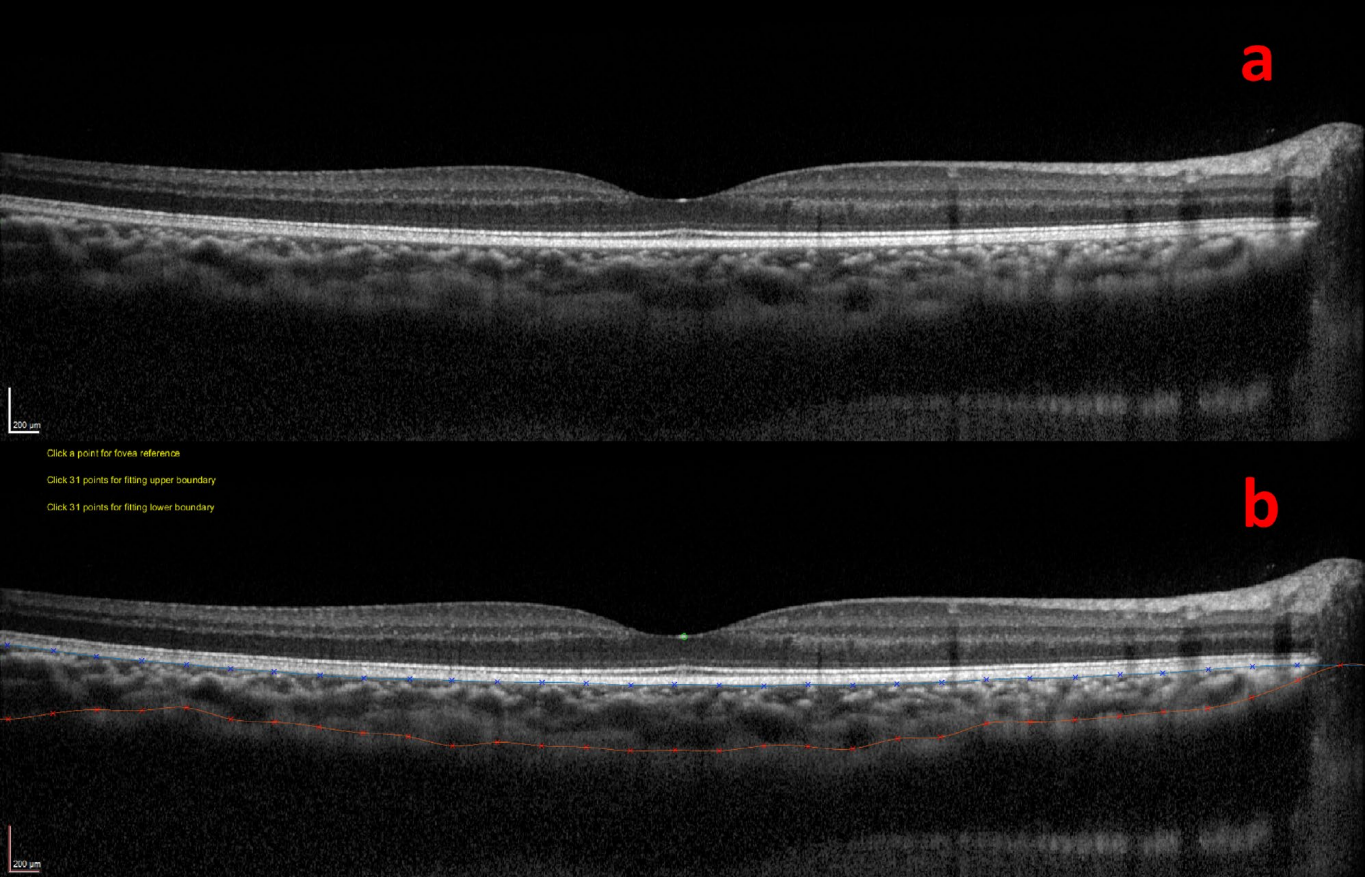
Measurement of choroidal thickness using the MATLAB software in spectral-domain optical coherence tomography (SD-OCT). (**a**) Presentation of the choroidal thickness before segmentation. (**b**) Manual segmentation of choroid by MATLAB software.

**Figure 2:**
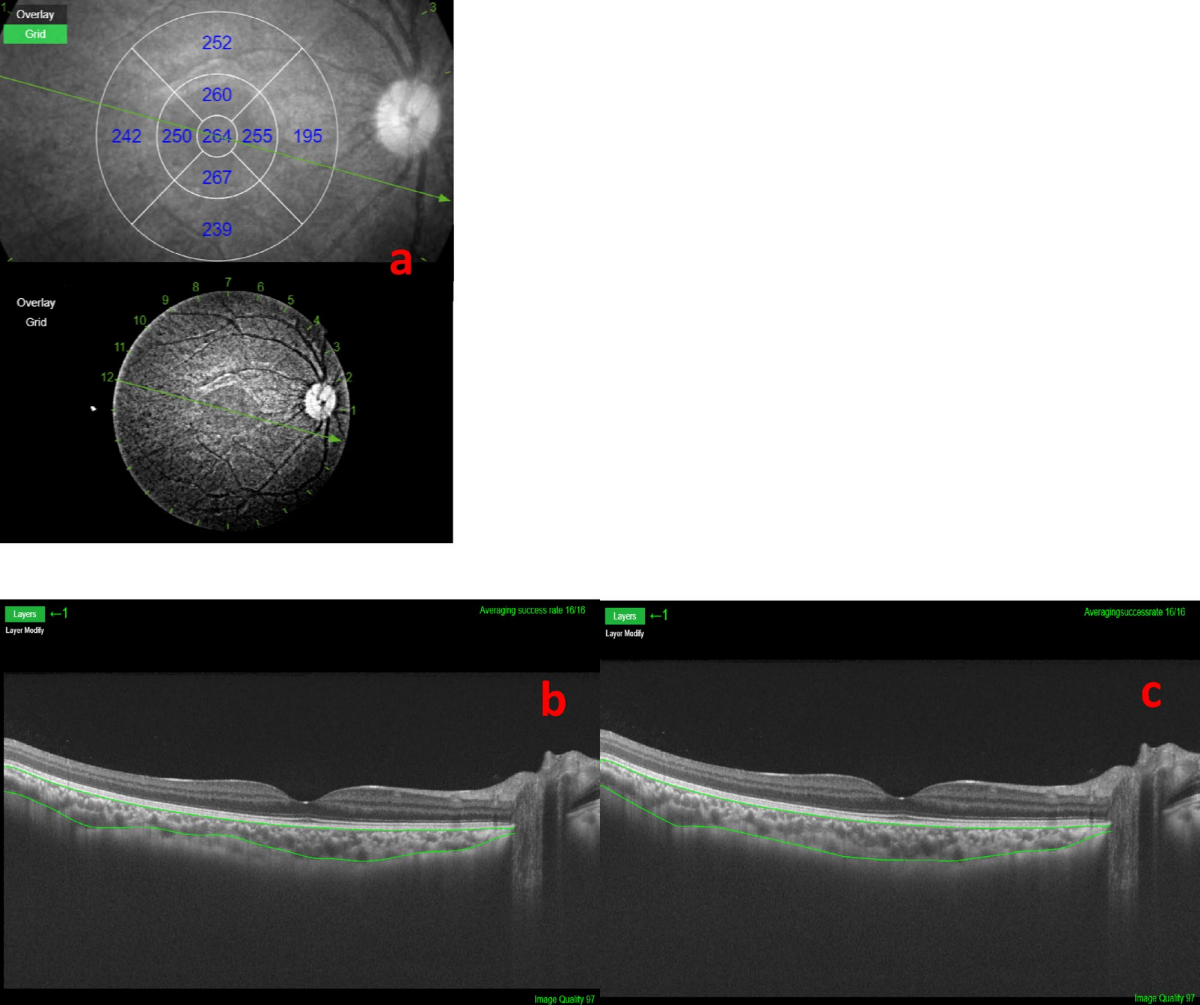
Measurement of choroidal thickness using the built-in software in swept-source optical coherence tomography (SS-OCT). (**a**) Presentation of the choroidal thickness with an Early Treatment Diabetic Retinopathy Study (ETDRS) grid (Diameters for central foveal circle, parafoveal circle and perifoveal circle are 1 mm, 3 mm and 6 mm, respectively). (**b**) Automatic segmentation of choroid by the built-in software before manual correction; (**c**) Manually corrected segmentation of choroid.

**Figure 3:**
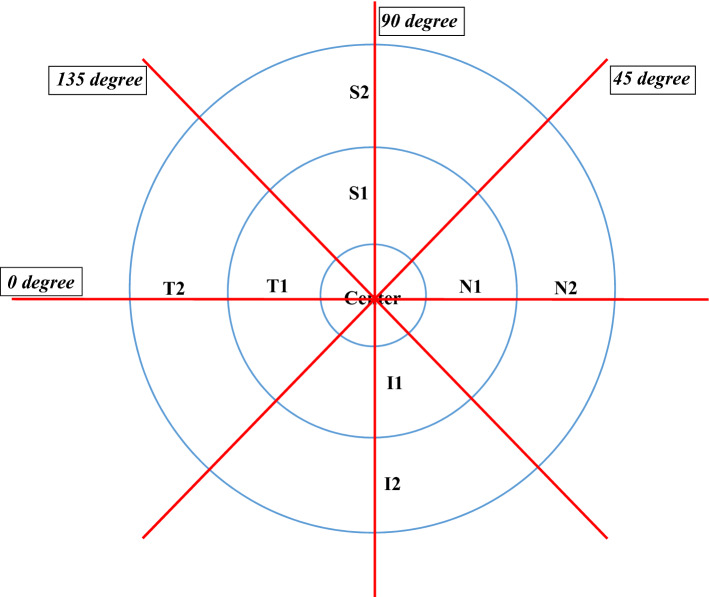
Schematic diagram showing axial scans from spectral-domain optical coherence tomography (SD-OCT) (Axial scan at 0°, 45°, 90° and 135°) in comparable with an Early Treatment Diabetic Retinopathy Study (ETDRS) grid (Diameters for central foveal circle, parafoveal circle and perifoveal circle are 1 mm, 3 mm and 6 mm, respectively). For SD-OCT, choroidal thickness of each regional sector was calculated from averaging thicknesses from 4 radial scans inside the central foveal circle for central foveal choroidal thickness (CFCT) (i.e. center area); or averaging thicknesses from 2 radial scans forming two linear edges of the annulus and 1 radial scan passing through the mid-point of the outer and inner circular edge for other regional sectors (i.e. S1, S2, I1, I2, T1, T2, N1 and N2).

We defined choroidal thickness as the perpendicular distance between the outer border of the retinal pigment epithelium and the inner border of the sclera^25^. Measurement of choroidal thickness of a study subject through SD-OCT and SS-OCT imaging is shown in Figs. 1 and 2 respectively. For SS-OCT, choroidal thickness map was presented using the Early Treatment Diabetic Retinopathy Study (ETDRS) grid^26^. Mean regional thickness was calculated for each of the 9 sectors in the ETDRS grid. For SD-OCT, we divided the whole region into 9 sectors as in the ETDRS grid for easy comparison with SS-OCT. Central foveal choroidal thickness (CFCT) was derived from the average thickness from 4 radial scans inside central foveal circle. Choroidal thicknesses in other regional sectors (i.e. S1, S2, I1, I2, T1, T2, N1 and N2) were derived from the average thicknesses from 2 radial scans forming two linear edges of the annulus and 1 radial scan passing through the mid-point of outer and inner circular (Fig. 3)^27^.

### Quality control

For quality control, all participants completed both OCT scans on the same day. We exclude participants if any of the OCT images in either device was in poor quality. Images were graded as poor quality if either the fovea or choroid-scleral junction was poorly visualised. All examiners were masked to all participant characteristics. To ensure reliability between the measurements, second measurement by the main trained examiner (C.O.L) and by another independent examiner (N.Y.) were performed. Both intra-grader and inter-grader reliability was high with intra-class correlation coefficient (ICC) of 0.969 and 0.906, or greater respectively (Supplementary Table S1 and S2). Furthermore, to distinguish the possibility that the segmentation method was not the primary cause of the measurement differences, we randomly selected 11% of the subjects (N = 12) and assessed the agreement of choroidal thickness measurement between semi-automated and manual segmentation for SS-OCT images. Comparison of choroidal thickness measurements between different segmentation methods is summarised in Supplementary Table S3. High agreement (ICC in central sector: 0.950) between semi-automated and manual segmentation for SS-OCT images was observed in all regional sectors (Supplementary Table S4).

### Statistical analysis

Data were analysed with SPSS (SPSS, Inc., Chicago, IL, version 24.0). Continuous parameters were expressed as mean ± standard deviation (SD). Bland–Altman plot was used to assess the agreement and limit of agreement. ICCs were analysed for the agreement between the two devices. A *P-*value of less than 0.05 was considered statistically significant.

Conversion equations were derived to translate measurement of choroidal thickness from SD-OCT into a predicted SS-OCT value. Outliers (N = 14) (defined as CFCT measurement difference by two devices is > 2 SDs away from the mean difference) were excluded from the derivation of conversion equations. Random half sample of the remaining participants (N = 48) were used to determine the conversion equations using linear regression and second half of the participants (N = 47) were used to validate the derived equations. Validity is evaluated by comparing observed and predicted SS-OCT measurements computed by the conversion equations.

## Results

### Demographics

After excluding 31 children with poor quality of OCT images, a total of 114 Chinese children, 57 (50.0%) boys and 57 (50.0%) girls, who successfully completed ophthalmic examinations were included for analysis. The mean age was 7.38 ± 0.82 years. Demographic characteristics, systemic and ocular parameters of the included participants are summarised in Table 1.

**Table 1:** Demographics of subjects.

n = 114	Total (mean ± SD)
Gender, female	n = 57 (50.0%)
Age, years	7.38 ± 0.82
Height, cm	123.51 ± 6.23
Weight, kg	24.00 ± 4.20
Spherical equivalent refraction (SER), diopters (D)	0.15 ± 1.40
Post-cycloplegic spherical power, diopters (D)	0.50 ± 1.43
Post-cycloplegic cylindrical power, diopters (D)	− 0.67 ± 0.81
Axial length, mm	23.05 ± 0.92

*SD* standard deviation, *SD-OCT* spectral-domain optical coherence tomography, *SS-OCT* swept-source optical coherence tomography.

### Choroidal thickness

By SD-OCT measurement, the CFCT was 273.24 ± 54.29 μm. Other regional sectors were inner superior (S1): 270.39 ± 50.91 μm; outer superior (S2): 263.25 ± 45.84 μm; inner inferior (I1): 266.94 ± 53.94 μm; outer inferior (I2): 256.57 ± 50.65 μm; inner temporal (T1): 283.41 ± 50.81 μm; outer temporal (T2): 286.88 ± 43.48 μm; inner nasal (N1): 252.39 ± 51.43 μm; and outer nasal (N2): 224.48 ± 47.20 μm. By SS-OCT measurement, the CFCT was 251.84 ± 47.12 μm. Other regional sectors were S1: 261.32 ± 48.93 μm; S2: 247.65 ± 44.54 μm; I1: 255.07 ± 48.17 μm; I2: 240.40 ± 46.40 μm; T1: 274.56 ± 45.21 μm; T2: 271.32 ± 40.11 μm; N1: 230.68 ± 56.99 μm; and N2: 183.90 ± 61.63 μm (Table 2).

**Table 2:** Comparison of choroidal thickness measurements between spectral-domain optical coherence tomography (SD-OCT) and swept-source optical coherence tomography (SS-OCT) in general and subgroup analysis classified by spherical and cylindrical power.

Mean ± SD	SD-OCT	SS-OCT	*P*-value	Mean difference
**Total participants (n = 114)**				
Centre, μm	273.24 ± 54.29	251.84 ± 47.12	< 0.001	21.40 ± 33.13
S1, μm	270.39 ± 50.91	261.32 ± 48.93	0.008	9.07 ± 36.07
S2, μm	263.25 ± 45.84	247.65 ± 44.54	< 0.001	15.60 ± 27.42
I1, μm	266.94 ± 53.94	255.07 ± 48.17	0.001	11.87 ± 37.47
I2, μm	256.57 ± 50.65	240.40 ± 46.40	< 0.001	16.17 ± 32.43
T1, μm	283.41 ± 50.81	274.56 ± 45.21	0.003	8.85 ± 31.38
T2, μm	286.88 ± 43.48	271.32 ± 40.11	< 0.001	15.56 ± 28.50
N1, μm	252.39 ± 51.43	230.68 ± 56.99	< 0.001	21.71 ± 43.41
N2, μm	224.48 ± 47.20	183.90 ± 61.63	< 0.001	40.57 ± 49.61

Spherical equivalent refraction (SER)				
**Myopia (SER ≤ − 0.50 D) (n = 28)**				
Centre, μm	244.83 ± 46.86	227.36 ± 43.05	0.022	17.48 ± 38.02
S1, μm	245.72 ± 42.69	236.68 ± 47.00	0.336	9.04 ± 48.88
S2, μm	241.48 ± 32.50	226.04 ± 42.87	0.038	15.44 ± 37.35
I1, μm	240.49 ± 47.23	236.00 ± 52.04	0.622	4.49 ± 47.56
I2, μm	233.34 ± 47.98	224.46 ± 50.33	0.341	8.87 ± 48.43
T1, μm	257.71 ± 46.93	251.96 ± 47.24	0.434	5.74 ± 38.27
T2, μm	263.49 ± 38.48	250.79 ± 43.27	0.049	12.71 ± 32.61
N1, μm	225.08 ± 39.75	209.57 ± 58.15	0.161	15.51 ± 56.97
N2, μm	201.28 ± 36.97	165.25 ± 62.43	0.006	36.03 ± 64.05
**Emmetropia (− 0.50 D < SER < + 0.50 D) (n = 33)**				
Centre, μm	275.68 ± 60.87	251.06 ± 50.27	0.001	24.62 ± 38.20
S1, μm	267.84 ± 51.11	257.12 ± 48.92	0.031	10.72 ± 27.31
S2, μm	260.02 ± 42.83	244.79 ± 43.29	< 0.001	15.23 ± 20.17
I1, μm	271.01 ± 60.78	251.7 ± 46.85	0.005	19.31 ± 36.93
I2, μm	258.48 ± 55.95	235.94 ± 45.24	< 0.001	22.55 ± 28.01
T1, μm	288.11 ± 57.04	275.09 ± 46.69	0.026	13.02 ± 32.09
T2, μm	286.54 ± 44.4	268.94 ± 33.78	0.002	17.60 ± 30.63
N1, μm	253.82 ± 56.47	223.55 ± 53.53	< 0.001	30.28 ± 31.35
N2, μm	222.88 ± 50.25	177.42 ± 58.65	< 0.001	45.46 ± 36.14
**Hypertrophia (SER ≥ + 0.50 D) (n = 53)**				
Centre, μm	286.73 ± 48.60	265.26 ± 42.43	< 0.001	21.47 ± 26.86
S1, μm	285.01 ± 50.30	276.96 ± 44.67	0.087	8.05 ± 33.55
S2, μm	276.77 ± 49.40	260.85 ± 42.07	< 0.001	15.92 ± 25.67
I1, μm	278.39 ± 48.68	267.25 ± 43.95	0.012	11.14 ± 31.17
I2, μm	267.66 ± 45.10	251.60 ± 42.75	< 0.001	16.06 ± 23.02
T1, μm	294.07 ± 44.48	286.17 ± 39.09	0.038	7.90 ± 27.00
T2, μm	299.44 ± 40.88	283.64 ± 37.90	< 0.001	15.80 ± 25.05
N1, μm	265.93 ± 48.73	246.28 ± 54.93	0.001	19.65 ± 41.63
N2, μm	237.73 ± 45.92	197.79 ± 60.84	< 0.001	39.93 ± 48.87

SD, standard deviation; SD-OCT, spectral-domain optical coherence tomography; SS-OCT, swept-source optical coherence tomography; Centre, central foveal; S1, inner superior; S2, outer superior; I1, inner inferior; I2, outer inferior; T1, inner temporal; T2, outer temporal; N1, inner nasal; N2, outer nasal.

For both devices, it was observed that choroidal thicknesses in parafoveal regional sectors were thicker than those in perifoveal regional sectors. Also, temporal sectors had the thickest choroid while nasal sectors had the thinnest choroid among all regional sectors.

### Comparison between SD-OCT and SS-OCT

**Figure 4:**
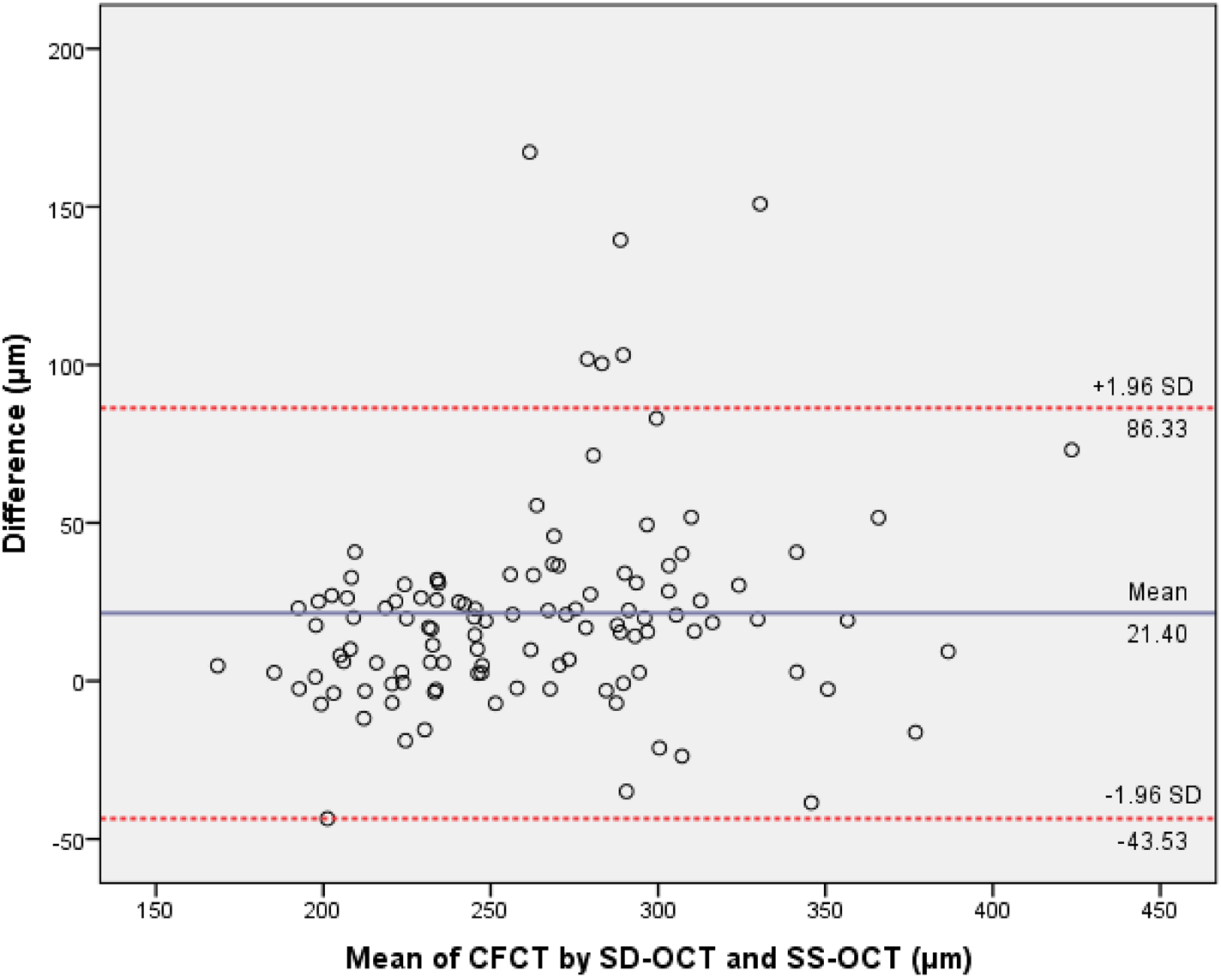
Bland–Altman plots comparing choroidal thickness measurements between spectral-domain optical coherence tomography (SD-OCT) and swept-source optical coherence tomography (SS-OCT) in healthy paediatric eyes.

There was good intra-class correlation for CFCT with an ICC of 0.840 (95% confidence interval (CI) 0.616–0.918) for absolute measurements and 0.881 (95% CI 0.828–0.918) for relative agreement (*P* < 0.001) (Table 3). Other regional sectors showed similar results with good ICCs, except for nasal sectors with slightly poor ICCs (N1: 0.774, 95% CI 0.595–0.864; N2: 0.636, 95% CI 0.150–0.815 for absolute measurements). Nevertheless, the mean difference for CFCT was 21.40 ± 33.13 μm and the thickness measurements from SD-OCT in all regional sectors were statistically significant thicker than that from SS-OCT (Table 2). Bland–Altman limit of agreement on the relative difference scale for SD-OCT/SS-OCT was 86.33 μm (Fig. 4). The derived conversion equation for translating SD-OCT CFCT measurement into SS-OCT was SS-OCT = 35.261 + 0.810 × SD-OCT. Validation showed satisfactory ICC (0.942, 95% CI 0.898–0.967). The predicted value fell within 10% of the actual SS-OCT CFCT measurement 94.7% of the time (Table 4).

**Table 3:** Intra-class correlation coefficients between spectral-domain optical coherence tomography (SD-OCT) and swept-source optical coherence tomography (SS-OCT) in general and subgroup analysis classified by spherical and cylindrical power.

Regional sectors	Absolute ICC (95%CI)	Relative ICC (95%CI)	*P*-value
**Total participants (n = 114)**			
Centre	0.840 (0.616–0.918)	0.881 (0.828–0.918)	< 0.001
S1	0.843 (0.869–0.893)	0.850 (0.783–0.896)	< 0.001
S2	0.871 (0.722–0.930)	0.899 (0.853–0.930)	< 0.001
I1	0.833 (0.746–0.889)	0.845 (0.775–0.893)	< 0.001
I2	0.849 (0.716–0.912)	0.875 (0.818–0.913)	< 0.001
T1	0.873 (0.810–0.915)	0.881 (0.827–0.918)	< 0.001
T2	0.837 (0.672–0.908)	0.869 (0.810–0.909)	< 0.001
N1	0.774 (0.595–0.864)	0.810 (0.724–0.869)	< 0.001
N2	0.636 (0.150–0.815)	0.743 (0.628–0.823)	< 0.001

Spherical equivalent refraction (SER)			
**Myopia (SER ≤ -0.50 D) (n = 28)**			
Centre	0.754 (0.454–0.888)	0.783 (0.530–0.899)	< 0.001
S1	0.579 (0.097–0.805)	0.579 (0.090–0.805)	0.014
S2	0.654 (0.271–0.838)	0.682 (0.314–0.853)	0.002
I1	0.709 (0.366–0.866)	0.703 (0.358–0.863)	0.001
I2	0.680 (0.314–0.852)	0.680 (0.308–0.852)	0.002
T1	0.804 (0.579–0.909)	0.802 (0.572–0.908)	< 0.001
T2	0.794 (0.552–0.905)	0.811 (0.593–0.913)	< 0.001
N1	0.505 (-0.039–0.767)	0.514 (-0.050–0.775)	0.033
N2	0.307 (-0.285–0.652)	0.362 (-0.380–0.705)	0.125
**Emmetropia (−0.50 D < SER < + 0.50 D) (n = 33)**			
Centre	0.825 (0.525–0.925)	0.867 (0.732–0.935)	< 0.001
S1	0.910 (0.809–0.957)	0.919 (0.837–0.960)	< 0.001
S2	0.913 (0.673–0.967)	0.942 (0.882–0.971)	< 0.001
I1	0.842 (0.629–0.927)	0.869 (0.735–0.935)	< 0.001
I2	0.874 (0.515–0.952)	0.918 (0.834–0.960)	< 0.001
T1	0.883 (0.750–0.944)	0.895 (0.788–0.948)	< 0.001
T2	0.781 (0.479–0.900)	0.822 (0.641–0.912)	< 0.001
N1	0.844 (0.275–0.946)	0.912 (0.821–0.956)	< 0.001
N2	0.737 (-0.133–0.913)	0.877 (0.751–0.939)	< 0.001
**Hypertrophia (SER ≥ + 0.50 D) (n = 53)**			
Centre	0.855 (0.495–0.940)	0.905 (0.836–0.945)	< 0.001
S1	0.853 (0.746–0.915)	0.858 (0.754–0.918)	< 0.001
S2	0.888 (0.707–0.947)	0.915 (0.853–0.951)	< 0.001
I1	0.861 (0.747–0.922)	0.873 (0.779–0.926)	< 0.001
I2	0.895 (0.680–0.954)	0.926 (0.872–0.957)	< 0.001
T1	0.877 (0.784–0.930)	0.884 (0.799–0.933)	< 0.001
T2	0.851 (0.622–0.930)	0.888 (0.805–0.935)	< 0.001
N1	0.778 (0.570–0.880)	0.808 (0.668–0.889)	< 0.001
N2	0.635 (0.111–0.827)	0.741 (0.552–0.851)	< 0.001

SD-OCT, spectral-domain optical coherence tomography; SS-OCT, swept-source optical coherence tomography; ICC, intra-class correlation coefficient of the two devices; Centre, central foveal; S1, inner superior, S2, outer superior; I1, inner inferior; I2, outer inferior; T1, inner temporal; T2, outer temporal; N1, inner nasal; N2, outer nasal.

**Table 4:** Conversion equations and validation data.

	Conversion equation (N = 48)	Validation (N = 47)			
		% of values within 10% of each other	Absolute ICC (95% CI)	*P*-value	Difference in means (μm)
Centre	SS-OCT = 35.261 + 0.810 × SD-OCT	94.7	0.942 (0.898–0.967)	< 0.001	3.57
S1	SS-OCT = 60.149 + 0.739 × SD-OCT	85.3	0.827 (0.707–0.899)	< 0.001	2.78
S2	SS-OCT = − 7.057 + 0.968 × SD-OCT	88.4	0.887 (0.806–0.935)	< 0.001	0.44
I1	SS-OCT = 46.750 + 0.783 × SD-OCT	88.4	0.832 (0.718–0.903)	< 0.001	1.57
I2	SS-OCT = 15.353 + 0.881 × SD-OCT	86.3	0.851 (0.747–0.914)	< 0.001	2.06
T1	SS-OCT = 57.869 + 0.768 × SD-OCT	91.6	0.883 (0.800–0.933)	< 0.001	1.87
T2	SS-OCT = 41.405 + 0.818 × SD-OCT	94.7	0.892 (0.814–0.938)	< 0.001	2.33
N1	SS-OCT = 20.396 + 0.827 × SD-OCT	81.1	0.729 (0.563–0.839)	< 0.001	5.53
N2	SS-OCT = − 11.260 + 0.837 × SD-OCT	75.8	0.606 (0.387–0.760)	< 0.001	0.68

SS-OCT, swept-source optical coherence tomography; SD-OCT, spectral-domain optical coherence tomography; ICC, intra-class correlation coefficient of the two devices; Centre, central foveal; S1, inner superior; S2, outer superior; I1, inner inferior; I2, outer inferior; T1, inner temporal; T2, outer temporal; N1, inner nasal; N2, outer nasal.

95 participants were included in the conversion equation analysis after exclusion of outliers (N = 19). Conversion equations were derived based on random half sample of participants (N = 48) and validation were based on the second half sample (N = 47).

### Sub-group analysis based on refractive status

The study children were further categorised according to their post-cycloplegic spherical equivalent refraction (SER), which was defined as the spherical diopters (D) plus one-half cylindrical diopters. Subjects are classified into (1) myopia (SER ≤ − 0.50 D) (n = 28); (2) emmetropia (− 0.50 D < SER < + 0.50 D) (n = 33); (3) hypertrophia (SER ≥ + 0.50 D) (n = 53). As shown in both OCT devices, children with myopia (CFCT from SD-OCT: 244.83 ± 46.86 μm; CFCT from SS-OCT: 227.36 ± 43.05 μm) tended to have a thinner choroid than emmetropic children (CFCT from SD-OCT: 275.68 ± 60.87 μm; CFCT from SS-OCT: 251.06 ± 50.27 μm) while children with hypertrophia tended to have a thicker choroid (CFCT from SD-OCT: 286.73 ± 48.60 μm; CFCT from SS-OCT: 265.26 ± 42.43 μm). All subgroups showed statistically significant difference in measured CFCT between the two OCTs (Table 2). While the inter-device correlation coefficients were excellent in both hypertrophic (CFCT: 0.855, 95% CI 0.495–0.940) (*P* < 0.001) and emmetropic eyes (CFCT: 0.825, 95% CI 0.525–0.925) (*P* < 0.001), but moderate in myopic eyes (CFCT: 0.754, 95% CI 0.454–0.888) (*P* < 0.001) (Table 3).

## Discussion

Our study shows generally satisfactory intra-device correlation coefficients of the widely commercially available SD-OCT and SS-OCT in normal Chinese children population, regardless of the refractive status. However, a statistically significant intra-device difference exists in the measurements. We also noted a marked superiority in the quality to visualise the choroid-sclera interface in SS-OCT (Fig. 2) compared to SD-OCT (Fig. 1), same as the finding previously reported in other studies^9,28^.

In a study of 35 healthy adult eyes, Matsuo et al.^11^ found a high intra-device correlation coefficient between SD-OCT and SS-OCT, which is consistent with our current study. Excellent correlation was also noted in other similar studies in normal adults^10,12,15,17^. In contrast to our findings, Matsuo et al.^11^ and Ikuno et al.^29^ revealed a thicker measured choroidal thickness from SS-OCT than from SD-OCT, which were both manually determined. The differences may be attributed to the automated segmentation of SS-OCT in our study. Zafar et al.^15^ observed that the mean subfoveal choroidal thickness measured manually by SD-OCT and SS-OCT was greater than that automatically determined SS-OCT. They reported that a considerably higher choroid-sclera interface was identified by automatic software. Nevertheless, in our current study, automatically segmented images were further inspected and the border was manually adjusted if required, so the difference may not be fully explainable.

Another postulation from an earlier study by Michalewski et al.^30^ was the difference in the measurement method. Manually determined SD-OCT choroidal thickness measurements were based on focal measurements whereas that of automatically determined SS-OCT were calculated from the average thickness in a circular area with a diameter of 1000 μm. As choroidal layer becomes thinner with the distance away from the fovea, focal measurements near fovea tend to overestimate the thickness and this may not be fully compensated by averaging focal measurements from different spots^30^.

In a paediatric study on choroidal thickness measurements by SS-OCT, Xiong et al.^31^ reported subfoveal choroidal thickness of 272 ± 61 μm, 283 ± 63 μm and 269 ± 61 μm for healthy children aged 6, 7 and 8 respectively. Similar studies done on Chinese school-age children also reported consistently thicker choroid than our subjects (251.84 ± 47.12 μm)^26,32–34^. Of note, our previous study showed that Hong Kong has a higher prevalence of myopia than other Chinese cities. The notable difference could be attributed to the more intensive pre-school education, along with frequent near-work activities and lack of outdoor time due to crowded living places in Hong Kong compared to other regions of China^22^. Given the widely accepted postulation of the negative relationship between choroidal thickness and myopia^26,35^, it is conceivable that the measured choroid is thinner in our study population.

In addition, we observed a statistically significant difference between the measurements obtained from the two devices, which differs from some previous studies on healthy adult eyes^10,15^. Hanumunthadu et al.^36^ reported thicker large choroidal vessel wall and thinner medium vessel wall at subfoveal area in children eyes compared to adult eyes. A denser and high-flow vascular network was also found in children eyes. We believe that the age-related variation in choroidal vasculature, in particular thicker and more crowded vessels, may result in a higher tissue density. It may affect the accuracy in automatic detection of borders of choroidal layer in SS-OCT. Furthermore, lowered penetrance may affect the visualisation of the lower borders, predominantly in SD-OCT with shorter wavelength.

As for topographical variation, our results are consistent with previous paediatric studies showing thickest and thinnest choroid layers in temporal and nasal regions respectively. Similarly, parafoveal sectors are thicker than perifoveal sectors as observed in these studies^26,31–34^. Our study further demonstrates poorer ICCs in nasal sectors compared to other sectors. We believe that the thin nasal choroidal layer results in a larger percentage error. Also, its proximity to the optic nerve may account for its higher variability compared to other regions. This also explains the less favourable validation data of conversion equations in nasal sectors.

In subgroup analysis, we observed similar ICCs in hypertrophic and emmetropic eyes, but poorer ICCs in myopic eyes in all regions (Table 3, Supplementary Figure S1). We believe that the inter-individual topographic variation of choroidal thickness during myopic growth may account for the difference. It is generally believed that choroid is stretched towards temporal direction when eyeball grows axially during myopic change^37–40^. Chui et al.^41^ proposed the ‘slippage’ hypothesis that difference in the rate and extent of stretching of tissue layers (retina, choroid and sclera) may result in slippage between ocular tissues during axial elongation. The ongoing process of scleral stretching gives rise to dynamic topographic asymmetry in choroidal thickness. In this regard, there is an inter-individual topographic variation of choroidal thickness depending on individual’s myopic progression. Unlike in the circular measurements by SS-OCT, focal measurements by SD-OCT may not truly reflect the variation and therefore results in a greater discrepancy. Furthermore, OCT was sometimes captured obliquely in myopic eyes. Although the central point still lies on the fovea, inaccurate perpendicular angle from choroid layer may also affect the ICCs.

Our study, to the best of our knowledge, is the first to compare the choroidal thickness measurements by two generations of OCT instruments in children. We directly compared measurements by different devices on the same subject.

Nevertheless, limitations of this study should be acknowledged. As mentioned above, SD-OCT choroidal thickness measurements were derived from focal measurements on different linear radial scans while that of SS-OCT were derived from regional measurements in a circular area. The discrepancy may be significant in children with greater topographical variation in choroidal thickness. Of note, most of these children are myopic, who are not the main focus of our current study.

In conclusion, we have shown a high consistency in choroidal thickness measurements between the two generations of OCT devices in healthy paediatric eyes. With a satisfactory ICC, it is justifiable to directly compare two sets of measurements for monitoring choroidal thickness changes in population-based epidemiological research study. Nevertheless, it should be noted that choroidal thickness measured by SD-OCT was statistically significantly greater than that measured by SS-OCT, especially in myopic children. Therefore, it is not recommended to interchange the two OCT-results when assessing disease progression in individual patient in clinical practice.

## Supplementary Information

**Supplementary Tables 1.**.

**Supplementary Figure S1.**.

## Data Availability

The datasets generated during and/or analysed during the current study are available from the corresponding author on reasonable request.
