# Supplementary material for: Comparison of choroidal thickness measurements between spectral domain optical coherence tomography and swept source optical coherence tomography in children
Source: Sci Rep. 2021 Jul 2;11:13749. doi: 10.1038/s41598-021-92980-9 (PMC8253773; doi:10.1038/s41598-021-92980-9)
Supplement: Supplementary file 1 — Supplementary Tables 1.. [file 41598_2021_92980_MOESM1_ESM.docx]

**Comparison of Choroidal Thickness Measurements between Spectral Domain Optical Coherence Tomography and Swept Source Optical Coherence Tomography in Children**

Chun On Lee^1^; Xiujuan Zhang^1^, PhD; Nan Yuan^1^, MPhil; Shumin Tang^1^, PhD; Li Jia Chen^1,2^, PhD; Carol Y Cheung^1^, PhD; *Jason C Yam^1,2,3^, FRCSEd

^1^Department of Ophthalmology and Visual Sciences, The Chinese University of Hong Kong, Hong Kong

^2^Department of Ophthalmology and Visual Sciences, Prince of Wales Hospital, Hong Kong

^3^Hong Kong Eye Hospital, Kowloon, Hong Kong

**TITLES AND LEGENDS TO TABLES**

Supplementary Table 1. Reliability with intra grader (11% of subjects N=12).

Supplementary Table 2. Reliability with inter grader (11% of subjects N=12).

Supplementary Table 3. Comparison of choroidal thickness measurements between different segmentation methods.

Supplementary Table 4. Reliability between semi-automated and manual segmentation for SS-OCT images.

**Supplementary Table 1. Reliability with intra grader (11% of subjects N=12).**

| **SD-OCT** | | |  | |
| --- | --- | --- | --- | --- |
| **Regional sectors** | **Absolute ICC (95%CI)** | **Relative ICC (95%CI)** | ***P*-value** | |
| Center | 0.982 (0.889-0.996) | 0.989 (0.961-0.997) | <0.001 | |
| S1 | 0.984 (0.929-0.996) | 0.987 (0.957-0.996) | <0.001 | |
| S2 | 0.988 (0.960-0.997) | 0.988 (0.959-0.997) | <0.001 | |
| I1 | 0.970 (0.893-0.991) | 0.973 (0.907-0.992) | <0.001 | |
| I2 | 0.985 (0.950-0.996) | 0.984 (0.946-0.995) | <0.001 | |
| T1 | 0.975 (0.880-0.994) | 0.982 (0.939-0.995) | <0.001 | |
| T2 | 0.976 (0.919-0.993) | 0.977 (0.922-0.994) | <0.001 | |
| N1 | 0.979 (0.927-0.994) | 0.982 (0.936-0.995) | <0.001 | |
| N2 | 0.969 (0.793-0.992) | 0.981 (0.933-0.994) | <0.001 | |
|  |  |  |  | |
| **SS-OCT** |  |  |  | |
| \| **Regional sectors** \| **Absolute ICC (95%CI)** \| **Relative ICC (95%CI)** \| ***P*-value** \| \| --- \| --- \| --- \| --- \| | | | |  |
| Center | 1.000 (0.999-1.000) | 1.000 (0.999-1.000) | <0.001 | |
| S1 | 0.999 (0.998-1.000) | 0.999 (0.998-1.000) | <0.001 | |
| S2 | 0.998 (0.992-0.999) | 0.997 (0.991-0.999) | <0.001 | |
| I1 | 0.999 (0.996-1.000) | 0.999 (0.996-1.000) | <0.001 | |
| I2 | 1.000 (0.999-1.000) | 1.000 (0.999-1.000) | <0.001 | |
| T1 | 0.998 (0.992-0.999) | 0.998 (0.992-0.999) | <0.001 | |
| T2 | 0.997 (0.991-0.999) | 0.997 (0.990-0.999) | <0.001 | |
| N1 | 0.999 (0.995-1.000) | 0.999 (0.996-1.000) | <0.001 | |
| N2 | 0.998 (0.992-0.999) | 0.998 (0.992-0.999) | <0.001 | |
|  |  |  |  | |

SD-OCT: spectral-domain optical coherence tomography; SS-OCT: swept-source optical coherence tomography; ICC: Intra-class correlation coefficient of two devices; Center: Central foveal; S1: Inner superior: S2: Outer superior; I1: inner inferior; I2: outer inferior; T1: Inner temporal; T2: Outer temporal; N1: Inner nasal; N2: Outer nasal.

**Supplementary Table 2. Reliability with inter grader (11% of subjects N=12).**

| **SD-OCT** | | |  | |
| --- | --- | --- | --- | --- |
| **Regional sectors** | **Absolute ICC (95%CI)** | **Relative ICC (95%CI)** | ***P*-value** | |
| Center | 0.973 (0.905-0.992) | 0.970 (0.897-0.992) | <0.001 | |
| S1 | 0.979 (0.929-0.994) | 0.978 (0.924-0.994) | <0.001 | |
| S2 | 0.950 (0.833-0.985) | 0.950 (0.827-0.986) | <0.001 | |
| I1 | 0.943 (0.800-0.984) | 0.938 (0.785-0.982) | <0.001 | |
| I2 | 0.941 (0.796-0.983) | 0.937 (0.781-0.982) | <0.001 | |
| T1 | 0.961 (0.870-0.989) | 0.963 (0.871-0.989) | <0.001 | |
| T2 | 0.906 (0.676-0.973) | 0.916 (0.710-0.976) | <0.001 | |
| N1 | 0.981 (0.937-0.995) | 0.981 (0.932-0.994) | <0.001 | |
| N2 | 0.950 (0.824-0.986) | 0.946 (0.811-0.984) | <0.001 | |
|  |  |  |  | |
| **SS-OCT** |  |  |  | |
| \| **Regional sectors** \| **Absolute ICC (95%CI)** \| **Relative ICC (95%CI)** \| ***P*-value** \| \| --- \| --- \| --- \| --- \| | | | |  |
| Center | 0.998 (0.992-1.000) | 0.999 (0.995-1.000) | <0.001 | |
| S1 | 0.995 (0.977-0.999) | 0.996 (0.985-0.999) | <0.001 | |
| S2 | 0.998 (0.992-0.999) | 0.998 (0.993-0.999) | <0.001 | |
| I1 | 0.997 (0.990-0.999) | 0.997 (0.990-0.999) | <0.001 | |
| I2 | 1.000 (0.999-1.000) | 1.000 (0.999-1.000) | <0.001 | |
| T1 | 0.929 (0.759-0.980) | 0.936 (0.778-0.982) | <0.001 | |
| T2 | 0.996 (0.985-0.999) | 0.996 (0.986-0.999) | <0.001 | |
| N1 | 0.998 (0.990-0.999) | 0.998 (0.994-0.999) | <0.001 | |
| N2 | 0.997 (0.991-0.999) | 0.998 (0.992-0.999) | <0.001 | |
|  |  |  |  | |

SD-OCT: spectral-domain optical coherence tomography; SS-OCT: swept-source optical coherence tomography; ICC: Intra-class correlation coefficient of two devices; Center: Central foveal; S1: Inner superior: S2: Outer superior; I1: inner inferior; I2: outer inferior; T1: Inner temporal; T2: Outer temporal; N1: Inner nasal; N2: Outer nasal.

**Supplementary Table 3. Comparison of choroidal thickness measurements between different segmentation methods.**

|  | **Mean±SD** | | | **Mean Difference±SD** | |
| --- | --- | --- | --- | --- | --- |
|  | **Manual SD-OCT** | **Semi-automated SS-OCT** | **Manual SS-OCT** | **Semi-automated SS-OCT vs Manual SS-OCT** | **Manual SD-OCT vs Manual SS-OCT** |
| **Center,μm** | 237.26±40.15 | 222.17±29.21 | 219.14±29.74 | 3.03±12.99 | 18.12±21.44 |
| **S1,μm** | 238.34±35.49 | 222.67±32.52 | 220.45±29.07 | 2.22±13.52 | 17.89±21.07 |
| **S2,μm** | 233.98±35.08 | 216.50±35.43 | 209.16±30.04 | 7.34±15.53 | 24.81±19.83 |
| **I1,μm** | 239.52±44.79 | 223.08±37.19 | 218.31±31.07 | 4.77±12.24 | 21.21±19.62 |
| **I2,μm** | 227.38±42.56 | 212.00±37.14 | 200.68±30.55 | 11.32±15.87 | 26.70±20.87 |
| **T1,μm** | 251.25±44.00 | 241.42±37.04 | 231.67±32.71 | 9.75±13.94 | 19.58±17.59 |
| **T2,μm** | 252.74±43.58 | 243.08±36.21 | 237.50±34.69 | 5.58±3.36 | 15.23±17.79 |
| **N1,μm** | 222.69±37.54 | 191.50±27.92 | 186.47±27.93 | 5.03±7.57 | 36.22±20.88 |
| **N2,μm** | 199.23±33.48 | 143.83±27.66 | 147.65±24.59 | -3.81±6.64 | 51.58±24.40 |

SD: standard deviation; SD-OCT: spectral-domain optical coherence tomography; SS-OCT: swept-source optical coherence tomography; Center: Central foveal; S1: Inner superior: S2: Outer superior; I1: inner inferior; I2: outer inferior; T1: Inner temporal; T2: Outer temporal; N1: Inner nasal; N2: Outer nasal.

**Supplementary Table 4. Reliability between semi-automated and manual segmentation for SS-OCT images.**

| **SS-OCT (Semi-automated vs Manual)** | | |  |
| --- | --- | --- | --- |
| **Regional sectors** | **Absolute ICC (95%CI)** | **Relative ICC (95%CI)** | ***P*-value** |
| Center | 0.950 (0.834-0.986) | 0.949 (0.823-0.985) | <0.001 |
| S1 | 0.952 (0.837-0.986) | 0.949 (0.825-0.985) | <0.001 |
| S2 | 0.933 (0.767-0.981) | 0.941 (0.794-0.983) | <0.001 |
| I1 | 0.965 (0.882 -0.990) | 0.967 (0.885-0.991) | <0.001 |
| I2 | 0.920 (0.634 -0.979) | 0.942 (0.800-0.983) | <0.001 |
| T1 | 0.943 (0.734 -0.985) | 0.959 (0.856-0.988) | <0.001 |
| T2 | 0.992 (0.638 -0.998) | 0.998 (0.992-0.999) | <0.001 |
| N1 | 0.975 (0.883-0.993) | 0.981 (0.935-0.995) | <0.001 |
| N2 | 0.980 (0.919-0.994) | 0.984 (0.943-0.995) | <0.001 |
|  |  |  |  |

SS-OCT: swept-source optical coherence tomography; ICC: Intra-class correlation coefficient of two devices; Center: Central foveal; S1: Inner superior: S2: Outer superior; I1: inner inferior; I2: outer inferior; T1: Inner temporal; T2: Outer temporal; N1: Inner nasal; N2: Outer nasal.
