# Supplementary material for: Comparison of choroidal thickness measurements between spectral domain optical coherence tomography and swept source optical coherence tomography in children
Source: Sci Rep. 2021 Jul 2;11:13749. doi: 10.1038/s41598-021-92980-9 (PMC8253773; doi:10.1038/s41598-021-92980-9)

**Comparison of Choroidal Thickness Measurements between Spectral Domain Optical Coherence Tomography and Swept Source Optical Coherence Tomography in Children**

Chun On Lee^1^; Xiujuan Zhang^1^, PhD; Nan Yuan^1^, MPhil; Shumin Tang^1^, PhD; Li Jia Chen^1,2^, PhD; Carol Y Cheung^1^, PhD; *Jason C Yam^1,2,3^, FRCSEd

^1^Department of Ophthalmology and Visual Sciences, The Chinese University of Hong Kong, Hong Kong

^2^Department of Ophthalmology and Visual Sciences, Prince of Wales Hospital, Hong Kong

^3^Hong Kong Eye Hospital, Kowloon, Hong Kong

**TITLES AND LEGENDS TO FIGURES**

Supplementary Figure 1. Measurement of choroidal thickness in a case of myopic subject. a) Spectral-domain optical coherence tomography (SD-OCT). b) Swept-source optical coherence tomography (SS-OCT).

**Supplementary Figure 1. Measurement of choroidal thickness in a case of myopic subject.** a) Spectral-domain optical coherence tomography (SD-OCT). b) Swept-source optical coherence tomography (SS-OCT).

**aa**


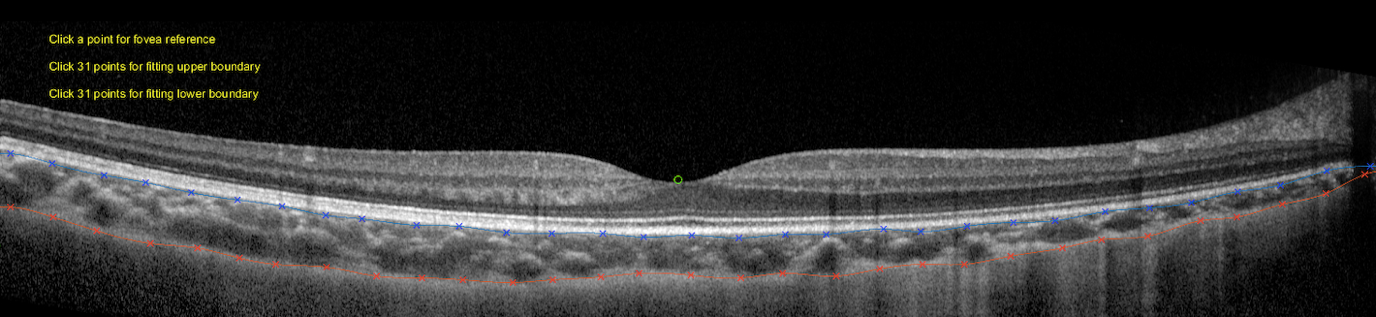


**ba**


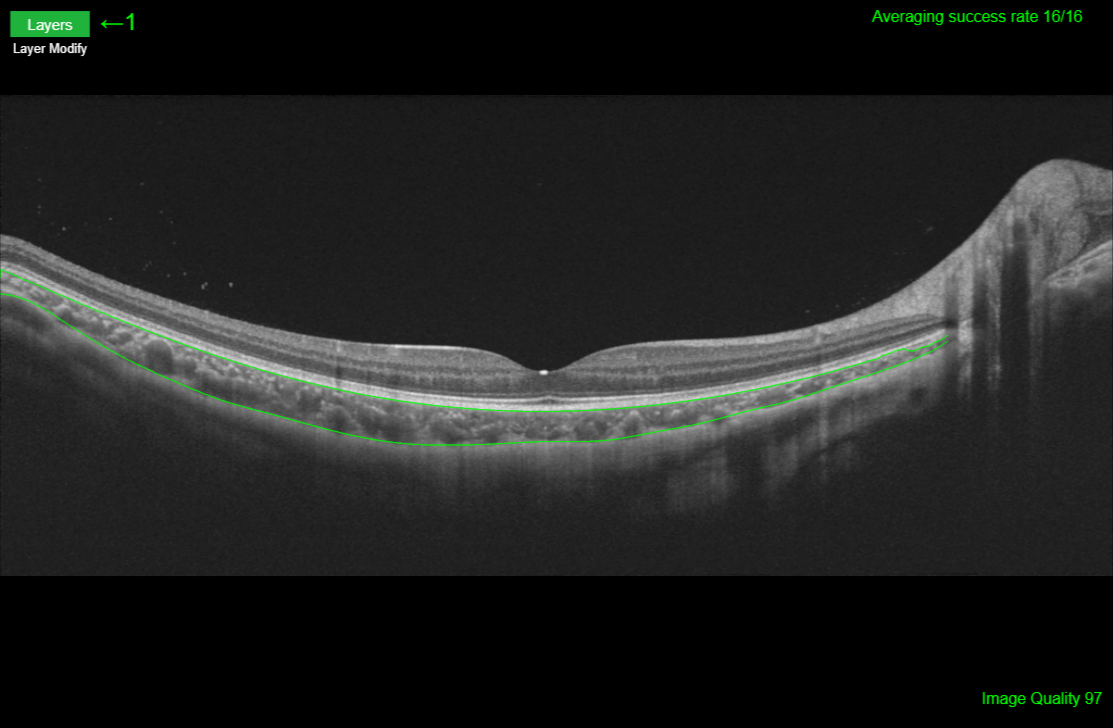

Supplement: Supplementary file 2 — Supplementary Figure S1.. [file 41598_2021_92980_MOESM2_ESM.docx]
